# Intersections of the Guiding Principles With Leadership Assessments

**DOI:** 10.1002/yd.70001

**Published:** 2025-08-17

**Authors:** Gayle Spencer, William Smedick

**Affiliations:** ^1^ Illinois Leadership Center University of Illinois at Urbana‐Champaign Champaign Illinois USA; ^2^ Leadership Studies Program, Whiting School of Engineering Johns Hopkins University Baltimore Maryland USA

## Abstract

The International Leadership Association's *General Principles for Leadership Programs* is a document designed to provide resources to create, redesign, and assess leadership programs. This article provides a broad introduction to the Venn diagram developed by the ILA Committee for the Advancement of Leadership Programs (CALP). It is intended to help leadership educators, taking their program context into account, introduce and explain how to provide the necessary depth needed for good program design. ILA's Advancement of Leadership Programs project works to advance the ongoing leadership learning efforts of the General Principles and Guiding Questions living documents on programmatic development and review. CALP has been charged with working with ILA members to develop the best ways to use these tools in our work. This article is intended to help you understand the many resources available when looking at the delivery of leadership programs. In addition to the General Principles, we provide other resources that can aid in creating and redesigning leadership programs.

## 1 Introduction

### 1.1 Project History and Current Charge

In the early 2000s, a group of ILA members began meeting at ILA's global conferences to discuss the need for a document that developed guidelines and standards for leadership education programs. A research agenda was proposed, and the ILA Board of Directors approved it in April 2005. ILA's *Guiding Questions: Guidelines for Leadership Education Programs* (ILA 2009) was published in 2009.

In 2019, there was an identified need to convene and build on the work done in the Guiding Questions document. ILA convened a General Principles Task Force to develop guidelines for academic curricular and co‐curricular leadership programs. The task force met, researched, and wrote a concept paper titled *General Principles for Leadership Programs* (ILA 2021). It was published in 2021.

The work draws from previous research and initiatives regarding the evolution of leadership learning across cultures and regions of the world. The general principles within the document serve as a foundation upon which those designing new programs or those with existing leadership programs can promote continuous quality improvement. In 2022, the ILA Board convened the Committee for the Advancement of Leadership Programs (CALP) to oversee and advance the ongoing global leadership learning efforts in this area. The project goals included:

- Continuing to coordinate efforts with globally minded and like‐minded assessment frameworks, such as the Carnegie Foundation's Elective Classification for Public Purpose, and the Council for the Advancement of Standards in Higher Education (CAS), among others.
- Continuing global conversations regarding the *General Principles* to ensure their relevance across sector, culture, and time.
- Piloting and testing the Higher Education *Guiding Questions* Conceptual Framework.
- Establishing a subcommittee to review the Higher Education *Guiding Questions* Framework and adapt it to meet the needs of leadership programs in the business sector.
- Seeking input from ILA members and other experts and sharing updates and refinements of the projects’ documents and white papers through online and onsite formats with the membership.
- Researching and developing resources for organizations to use in the development of leadership programs.
- Continuing to document the project's progress and annually inform the ILA Board of the work accomplished.

As this list is comprehensive, the goal of working on all the bullet points is to enable ILA to have a document and a concept to use that is fluid and can be applied by the entire membership of ILA. The CALP group also recognized this document needed to be able to be applied to situations outside of the United States and in disciplines other than higher education.

## 2 Development of the Venn Diagram

**FIGURE 1:**
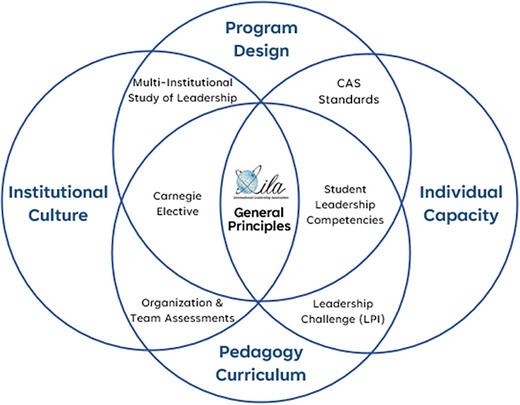
Committee for the Advancement of Leadership Programs Venn Diagram. Reprinted with permission from the Committee for the Advancement of Leadership Programs, 2024.

As the CALP group began to talk about connecting the General Principles with leadership assessments and tools that already existed, Rian Satterwhite from the University of Nevada‐Las Vegas developed a Venn diagram that helped us to visualize the connections between the ILA guiding principles and other assessments, instruments, and tools that help leadership educators in their work. At the core of the Venn diagram was the ILA General Principles. As discussions around this diagram continued, there were four components on the diagram: individual capacity, pedagogy curriculum, institutional culture, and program design (as depicted in Figure 1).

Next, we will describe and explain the four components: individual capacity, pedagogy curriculum, institutional culture, and program design. All the items in each component will also be listed. Since these items are in multiple components, a full explanation of each item, assessment, or tool will be discussed after all four components have been discussed.

## 3 The Four Components

### 3.1 Individual Capacity

“Individual capacity refers to a person's ability to take personal responsibility for their role, transform their attitudes and behaviors, and develop new skills and perspectives to contribute effectively to collective goals” (United Nations Development Programme 2006, 4). Other tools that address individual development and are listed in this component are the CAS Standards and Guidelines for Leadership Education and Development (CAS 2024), Student Leadership Competencies (Seemiller 2013), and the Leadership Challenge Leadership Practices Inventory (LPI) (Kouzes and Posner 2023b).

### 3.2 Pedagogy Curriculum

Pedagogy is referenced in the General Principles Concept Paper (ILA 2021) and regarded as describing how we can enhance student learning, and evidenced‐based practices, to enable learners’ demonstration of leadership knowledge, attitudes, skills, and aspirations. Pedagogy is the “how” we teach, which would include teaching strategies, learning activities, and assessment methods. When looking at curriculum, it is the specific subject matter, knowledge, and skills that we intend to teach. It is what we expect the students to learn.

The tools that are included in this category are Student Leadership Competencies (Seemiller 2013), the Leadership Challenge LPI (Kouzes and Posner 2023b), organization and team assessments, as well as the Carnegie Elective Classification for Leadership for Public Purpose (n.d.).

### 3.3 Institutional Culture

Also known as organizational culture, this is the way an institution incorporates its values, beliefs, and practices. A good culture can foster belonging and innovation, and improve engagement and retention of students, faculty, and staff. The tools that are included in this category are the Multi‐Institutional Study of Leadership (MSL), organization and team assessments, as well as the Carnegie Elective (n.d.).

### 3.4 Program Design

Program design is the process used to develop a structured plan for a program and usually identifies needs, defines goals, develops strategies, and designs the program's structure. Other tools that address program design and are listed in this category are the CAS Standards and Guidelines for Leadership Education and Development (CAS 2024), Student Leadership Competencies (Seemiller 2013), Dugan et al. (2013), and the Carnegie Elective (n.d.).

## 4 Discussion of Tools Referenced in the Venn Diagram

### 4.1 Carnegie Elective

With a clear mandate to influence higher education leadership programs, the Doerr Institute at Rice University hosted a working meeting of 30 scholars and practitioners in January 2020. At its conclusion, an agreement was made that a concise classification system for leadership education would help embed leadership education as a core function for US higher education. Furthermore, leadership education serves a public good purpose.

Over the next 5 years (2020–2025) a pilot program was launched that included a diverse group of higher education types (public, private, and service academies). The classification system was implemented at these founding institutions, and lessons were learned with adjustments made. The core question for the pilot was, “What would an institution that is committed to leadership for public purpose look like, act like, and be like?” With changes made, the number of institutions involved grew, and the benefit of achieving a Carnegie Classification became desirable and evident.

Colleges and universities that have invested in the Carnegie program have successfully expanded their leadership programs. This expansion allowed institutions the comfort of having the knowledge that they meet a high standard with the purposeful emphasis on public service. With this knowledge the faculty and staff committed to the Carnegie Classification program have direction and vision for their leadership programs. Institutions demonstrate commitment to effective leadership for public purpose in the learning, teaching, service, and research mission of their institution by:

- Embedding leadership for public purpose in the culture, structure, policies, and practices of the institution.
- Cultivating the knowledge, skills, and dispositions necessary for effective and ethical leadership in all institutional stakeholders.
- Developing leaders to meet complex challenges in their careers, their communities, and the broader society.
- Advancing the scholarly understanding and professional practice of leadership for public purpose; and
- Measuring the impact of their efforts (individually, programmatically, and institutionally) toward developing leaders for public purpose.

The inaugural year for the Carnegie Elective Classification for Leadership Public Purpose was 2024, and Table 1 lists the universities that have this classification:

**TABLE 1:** Carnegie Elective Classification for Leadership Public Purpose Universities.

Arizona State University	Boise State University	California State University, Fresno	Claremont McKenna College	Creighton University
East Carolina University	Florida International University	Fort Hays State University	Gettysburg College	James Madison University
Miami Dade College	Montclair State University	Oklahoma State University	Oral Roberts University	Rice University
Saint Peter's University	San Antonio College	Simmons University	University of Cincinnati	University of Portland
United States Coast Guard Academy	United States Naval Academy	Uniformed Services University of the Health Sciences	Valparaiso University	Wartburg College

*Note*: The next cycle will be in 2027, and applications must be secured by January 9, 2026.

### 4.2 CAS Standards

The Council for the Advancement of Standards in Higher Education (CAS) was founded in 1979 to create standards of professional practice that further the development of environments that foster student learning and development. Each edition includes a contextual statement as well as the Standards and Guidelines for Leadership Education and Programs (Spencer and Smedick 2020). Edition 11.1 of the CAS Professional Standards for Higher Education was released in January 2024.

Contextual statements are the “…introduction to a set of CAS Standards that offers sufficient background and perspective on the functional area to assist in understanding and applying the standards and guidelines. In addition, they provide context to the history, foundational principles, and current issues that influence the functional area standards” (CAS 2024, 1185–86).

All CAS standards and guidelines have the same 12 common criteria categories, which are commonly referred to as general standards. These categories apply to all functional areas, no matter the area of focus. There are also five guiding principles that overarch the parts. The twelve areas are as follows:

**Guiding Principle: Students and Their Environments**. Part 1, *Mission*, identifies the purpose and essential characteristics of the functional area. Part 2, *Program and Services*, describes how the area is structured and what it does. Part 3, *Student Learning, Development, and Success*, is related to how the area contributes to student growth processes, as well as how the given approach aligns with student learning and development models. Part 4, *Assessment, includes* how the area conducts, analyzes, and uses data and information gathering techniques and practices for ongoing improvement.

**Guiding Principle: Advocating for Diverse, Equitable, and Inclusive Communities**. Part 5, *Access,Diversity, Equity, Inclusion*, and Justice, outlines the area's role in advancing and maintaining access, diversity, equity, inclusion, and justice in the workplace and educational environments.

**Guiding Principle: Organization, Leadership, and Human Resources**. Part 6, *Leadership* is concerned with how the leaders of the area advance the work of the functional area. Part 7, *Human Resources*, outlines who are employed (e.g., professional staff, graduate students, student employees, volunteers), qualifications or credentials necessary, employment practices, onboarding, training, and development. Part 8, *Collaboration and Communication*, identifies key collaborators and partners, internal and external, with whom the area should consult or engage.

**Guiding Principle: Ethical Considerations**. Part 9, *Ethics, Law, and Policy*, includes standards for ethical practice, possible key legal issues, and policies and procedures critical to the area of work.

**Guiding Principle: Learning‐Conducive Structures, Resources, and Systems**. Part 10, *Financial Resources*, covers important fiscal considerations, including how to fund financial planning and accounting processes to consider. Part 11, *Technology*, depicts the role of technology in the area, including needs, ways to engage users, and management of information technologies. Part 12, *Facilities and Infrastructure*, details facilities, equipment, space, and other infrastructure needs that may be necessary for the area.

All functional areas have specialty standards and guidelines. All standards use the verbs “must” and “shall,” designating whether the statement that follows is an imperative or a suggestion, and appear in bold print to be quickly identified. Guidelines are intended to provide suggestions and illustrations that can assist in establishing programs and services that more fully address the needs of students. They appear in regular font and use the verbs “should” and “may.”

The beauty of the CAS standards is the multitude of ways they can be enhanced to improve or develop a program. With twelve areas, you can use one or all when looking to use the standards. They can be useful when designing news programs and services; they can help to focus time, energy, and resources; devise staff development activities; guide strategic planning; measure program and service effectiveness; assist with onboarding of new staff members; or create and assess learning and development outcomes.

The CAS standards and guidelines provide criteria that outline, guide, and ground planning. The Mission and Programs and Services sections can be helpful to specify goals and components. It can be helpful for providing understanding of what is needed to meet the essentials for programs and services. Finally, the standards and guidelines identify the scope of functions essential for comprehensive student leadership and leadership education and development programs.

When working on strategic planning, a CAS program review would be instrumental when developing a strategic plan as well as an assessment plan. When conducting a self‐assessment, it is helpful to use the CAS Self‐Assessment Guide (SAG). The SAG provides an effective workbook/format for evaluation, self‐assessment, and institutional reviews.

### 4.3 Student Leadership Competencies

“Leadership competencies are knowledge, values, abilities, and behaviors that help an individual contribute to or successfully engage in a role or task” (Seemiller 2013, xv). This work started on the premise that if we were going to help students adequately prepare for careers, we should understand the competencies necessary to be effective in their work.

There are 60 competencies that were developed and can serve as a framework for designing programs or courses, a blueprint for curriculum development and delivery, a foundation for intentional assessment of learning, and recognition of growth and development. There are six different learning domains that guide how each competency can be taught and assessed.

#### 4.3.1 Level 1

- **Significance**: Value of utilizing the competency. Leadership program offerings should include meaning‐making for students.
- **Motivation**: Motivation to utilize the competency. Intrinsic and extrinsic motivation should be distinguished in leadership programs. The ability for leaders to appeal to followers' values and align values as well as morals helps leaders succeed. Sessions designed to help students identify and commit to their personal values are encouraged.
- **Efficacy**: Belief in one's own ability to utilize the competency. Helping student leaders understand and have confidence in their ability to lead should be included in leadership development program offerings.

#### 4.3.2 Level 2

- **Cognition**: Understanding of the competency. As in other models discussed throughout this article, a key component of leadership education should include critical thinking skills.
- **Proficiency**: Skills to utilize the competency. A commitment to increasing a leader's skills and competencies can be an important element of a robust leadership development program. Helping students identify those skills that can be enhanced and having them commit to a strategic plan to increase competency will be helpful.

#### 4.3.3 Level 3

- **Performance**: Utilizing the competency. Providing experiential opportunities for students to use these competencies in their class projects and student and community organizations will allow students to learn from their success and mistakes. A tolerance for learning from failure is an important leadership trait. Seemiller (2013) also distributed 60 competencies into eight categories.
- **Learning and Reasoning**: In order to enhance critical thinking skills, students are encouraged to learn and are provided sessions on research, empathy, systems thinking, idea generation, decision‐making, and others.
- **Self‐Awareness and Development**: Leadership styles, CliftonStrengths (Gallup 1999). and specific leadership competencies are ways to help students with self‐awareness and increase their leadership competencies.
- **Interpersonal Interaction**: Communication skills and emotional intelligence are among the competencies that should be targeted in programs and sessions.
- **Group Dynamics**: The ways in which groups develop in positive ways and providing intervention strategies when groups are not functioning well, should be emphasized in leadership development programs. Some examples include Tuckman's team development model (Tuckman 1965), Lencioni's five dysfunctions of teams (Lencioni 2002), and Google's Aristotle Project (Google n.d.), which are tried and true models.
- **Civic Responsibility**: In many ways, civic responsibility answers the question “leadership for what?” Experiential programs that engage students in communities for public good are encouraged. Social innovation and entrepreneurship programs are ways faculty and staff can engage students and enhance their civic responsibility.
- **Communication**: Communication skills are vital to effective leadership. Opportunities to enhance verbal, written, and even nonverbal communication are encouraged.
- **Strategic Planning**: The idea that leadership and management competencies should intersect emerges with developing competencies related to effective strategic planning.
- **Personal Behavior**: Helping students understand that their behavior needs to reflect their morals and values is a core element for leadership education.

An example of an institution that developed a comprehensive multi‐level model for campus‐based leadership education is the University of Illinois at Urbana‐Champaign. This model is based on four classifications related to teaching, learning, and evaluation of leadership capacity. The model consists of a philosophy of leadership, a four‐level competency‐based model, a blueprint for where these skills and competencies can be learned, practiced, and developed, and specific plans for assessing and evaluating leadership development (Rosch et al. 2017).

This competency‐based model was developed as a result of focus groups with faculty, staff, and students on campus. The three questions asked were: What leadership skills, values, or attributes will a 2021 graduate of the University of Illinois demonstrate? As we look to develop leaders for the 21st century, how should we approach cultivating leadership in our students? How would you compare and contrast the skills, values, and attributes we discussed with the model of leadership the Illinois Leadership Center has been using? Data analysis provided both themes for competencies and approaches and practices to cultivate leadership. Table 2 displays the four levels of practice (personal/self, interpersonal/team, organization, and community/society). There are 21 competencies, which are also described to help understand each competency. (Illinois Leadership Center, n.d).

**TABLE 2:** Illinois Leadership Model.

Personal/self	Interpersonal/team	Organization	Community/society
Self‐knowledge	Common purpose	Change management	Human dignity
Self‐management	Communication	Diversity advocacy	Social justice
Reflection	Relationship management	Systems thinking	Global competence
Empathy	Group dynamics	Innovation	Service‐minded
Openness	Followership	—	Sustainability
Integrity	Cultural competency	—	—

### 4.4 Leadership Challenge and the LPI

The Leadership Challenge (Posner and Kouzes 2023a) and the accompanying leadership workbook and assessments provide leadership educators with a framework for leadership development as well as specific strategies to employ when teaching and providing sessions for students in leadership programs. There are specific student inventories that are called the Student LPI 360 and the Student LPI Self. The elements of the Leadership Challenge include:

**Model the Way**: Do what you say you will do. Leaders' ability to role model leadership behaviors for followers is discussed and pedagogy to enhance one's ability to do so is included.
**Inspire a Shared Vision**: See and share exciting possibilities for the future.
Motivating followers through a vision that inspires and is both a stretch and attainable is the emphasis of this principle.
**Challenge the Process**: Empower others to take initiative and experiment.
Leadership education programs should provide change management constructs to help leaders inculcate change and innovation in an organization's culture.
**Enable Others to Act**: Foster Collaboration and build up others. Leadership style models such as the situational approach encourage leaders to adapt their leadership style to the needs of followers.
**Encourage the Heart**: Celebrate others by showing gratitude. Intrinsic motivation is a powerful way for leaders to inspire and sustain motivation. In the Leadership Challenge, understanding and employing heartfelt emotion is explored and practiced in this important construct.

An example of an institution that has developed a program that uses The Leadership Challenge is Johns Hopkins University's first‐year student seminar beginning in 2013. With a series of guest speakers that include a faculty/staff member and an experienced student leader, each session reflects one of the Leadership Challenge principles. A final session is a community service element that attempts to bring all the elements together in order to look at the sum of the model as opposed to each part. The elements of the Leadership Challenge class for first‐year students at Johns Hopkins University include:

Course Schedule:

**Week 1**

Theme: The Practices and Commitments of Exemplary Leaders

Course introduction
Relationship building
Review of content areas (five fundamental practices)
Class expectations
Student activities fair strategies

**Week 2**

Experiential Component: Teambuilding at the O'Connor Recreation Center

**Week 3**

Theme: Challenging the Process

Motivation
Goal setting; academic and extracurricular
Visioning success
Using personal insight (complete SLPI for homework)

**Week 4**

Experiential Component: Career Services

**Week 5**

Theme: Inspiring a Shared Vision

Hopkins history with Jim Stimpart, university archivist
SLPI discussion
CSC and Health Services staff and student presentation
Experiential component: Hopkins history/founders discoveries at MSE Library

**Week 6**

Theme: Enabling Others to Act

Building trust
Improv exercises

**Week 7**

Experiential Component: Hopkins Shuttle Trip and Tours of Peabody and Medical School Campus

**Week 8**

Theme: Modeling the Way

Values discussion
Presentation by Deans Boswell, Shephard, and Sanchez

**Week 9**

Experiential Component: Faculty Interaction Program

(Optional DC trip on the weekend)

**Week 10**

Theme: Encouraging the Heart

Positive psychology presentation

**Week 11**

Experiential Component: Presentation by varsity coaches/student athletes and attend a varsity athletic event

**Week 12**

Theme: Commitments

Reflection paper due

**Week 13**

Experiential Component: Exit interviews with upper‐class students and staff

### 4.5 MSL

The MSL is an international research program focused on understanding the influences that shape socially responsible human development capacities and other leadership‐related outcomes (e.g., efficacy, cognitive skills, and resiliency). The MSL is designed to support a variety of audiences in documenting organizational and programmatic impact as well as optimizing the return on investments associated with learning initiatives.

The conceptual framework is based on Astin's (1993) input‐environment‐output (I‐E‐O) college impact model. The theoretical framework began with the social change model of leadership development (Astin and Astin 1996). The MSL was first administered in the spring of 2006. Some of the MSL's key findings are as follows:

1. **Socially Responsible Leadership**. Students showed growth in leadership rooted in ethics, collaboration, and a commitment to the common good.
2. **Mentorship**. Supportive relationships with mentors, especially faculty and staff, boosted students’ leadership development and confidence.
3. **Campus Climate**. A welcoming and inclusive environment on campus was key in shaping students’ leadership identity and development.
4. **Cognitive Skills**. Leadership experiences helped students sharpen their critical thinking, decision‐making, and problem‐solving abilities.
5. **Resilience and Hope**. Students involved in leadership programs were more resilient and had a more optimistic outlook on their future (Washburn University 2018).

### 4.6 Organization and Team Assessments

The ability to work effectively in team‐based organizations cannot be overstated. Leadership education programs are positioned to lead higher education institutions in developing pedagogy for students to learn and develop team skills. Some team‐based leadership constructs include models developed by Tuckman (1965) and Lencioni (2002).

Leadership educators can find utility in using Tuckman's stages of group development, which were developed in 1965 and revised in 1977. Tuckman provides a framework for leaders to understand positive team development as well as strategies that leaders may use to move a team to the highest levels of accomplishment. The five stages are:

**Forming**. This is where a group starts to meet.
**Storming**. This is the stage where there can be conflict and tension.
**Norming**. The group begins to establish how they will function and the expectations of its members.
**Performing**. The group starts to run in an effective manner.
**Adjourning**. The group has accomplished its task or tasks, and it finished with its work.

### 4.7 Lencioni's Five Dysfunctions of a Team

The “why” teams fail is the research question Patrick Lencioni (2002) poses to leadership educators and practitioners. The description in the book includes a case study for critical thinking discussion in leadership sessions. In an accompanying field guide, Lencioni (2005) provides strategies to use to prevent the dysfunctions or address a concern that develops. There is also a workbook (Lencioni 2012) that intact groups can use. The five dysfunctions include:

**Absence of Trust**. Fear of being open creates distrust.
**Avoidance of Conflict**. Not having healthy debate hinders progress.
**Lack of Commitment**. Having unclear goals can lead to weak buy‐in.
**Lack of Accountability**. Without accountability, tasks can remain unfinished.
**Inattention to Results**. Personal achievements outweigh team success.

## 5 Conclusion

For leadership educators who desire to improve their programs and academic offerings, this article provides resources to build, frame, and ultimately provide students with the skills to advance their careers and lead their communities. Leadership skills at their core are designed to enhance human skills, available to all. In addition, leadership programs should be designed to continuously improve. The resources included in this chapter can assist with formal assessments internally and externally.

While there are many items, assessments, or tools that are discussed in the article, at the core of the Venn diagram are the ILA general principles. The principles are designed to guide leadership programs whether they are in an academic or co‐curricular setting. The General Principles are more general and will help you think about leadership development at a broader level. The other tools and assessments discussed can help with more specific methods to shape your program and its offerings. This article has been designed to be a smorgasbord of resources, from which the reader can then pick and choose what may work best for them. They should be used or considered because they can provide a structured approach to quality improvement, can be used in various contexts and at varying programmatic levels, as well as provide international contexts and perspectives as you plan your programs and services, making sure that learners are at the core of the experiences.
